# Transcriptomics analysis reveals potential mechanisms underlying mitochondrial dysfunction and T cell exhaustion in astronauts’ blood cells in space

**DOI:** 10.3389/fimmu.2024.1512578

**Published:** 2025-01-20

**Authors:** Maria Moreno-Villanueva, Luis E. Jimenez-Chavez, Stephanie Krieger, Liang-Hao Ding, Ye Zhang, Adriana Babiak-Vazquez, Mark Berres, Sandra Splinter, Kristen E. Pauken, Brian C. Schaefer, Brian E. Crucian, Honglu Wu

**Affiliations:** ^1^ National Aeronautics and Space Administration, Johnson Space Center, Houston, TX, United States; ^2^ Department of Sport Science, University of Konstanz, Konstanz, Germany; ^3^ College of Medicine, University of Central Florida, Orlando, FL, United States; ^4^ KBR, Houston, TX, United States; ^5^ Department of Radiation Oncology, University of Texas Southwestern Medical Center, Dallas, TX, United States; ^6^ National Aeronautics and Space Administration, Kennedy Space Center, Cape Canaveral, FL, United States; ^7^ Bioinformatics Resource and Gene Expression Center, University of Wisconsin, Madison, WI, United States; ^8^ Department of Immunology, The University of Texas MD Anderson Cancer Center, Houston, TX, United States; ^9^ Department of Microbiology and Immunology, Uniformed Services University, Bethesda, MD, United States

**Keywords:** spaceflight, transcriptomics, astronauts’ health, mitochondria, immune dysfunction, telomere lengthening

## Abstract

**Introduction:**

The impact of spaceflight on the immune system and mitochondria has been investigated for decades. However, the molecular mechanisms underlying spaceflight-induced immune dysregulations are still unclear.

**Methods:**

In this study, blood from eleven crewmembers was collected before and during International Space Station (ISS) missions. Transcriptomic analysis was performed in isolated peripheral blood mononuclear cells (PBMCs) using RNA-sequencing. Differentially expresses genes (DEG) in space were determined by comparing of the inflight to the preflight samples. Pathways and statistical analyses of these DEG were performed using the Ingenuity Pathway Analysis (IPA) tool.

**Results:**

In comparison to pre-flight, a total of 2030 genes were differentially expressed in PBMC collected between 135 and 210 days in orbit, which included a significant number of surface receptors. The dysregulated genes and pathways were mostly involved in energy and oxygen metabolism, immune responses, cell adhesion/migration and cell death/survival.

**Discussion:**

Based on the DEG and the associated pathways and functions, we propose that mitochondria dysfunction was caused by constant modulation of mechano-sensing receptors in microgravity, which triggered a signaling cascade that led to calcium overloading in mitochondria. The response of PBMC in space shares T-cell exhaustion features, likely initiated by microgravity than by infection. Consequences of mitochondria dysfunction include immune dysregulation and prolonged cell survival which potentially explains the reported findings of inhibition of T cell activation and telomere lengthening in astronauts.

**Conclusion:**

Our study potentially identifies the upstream cause of mitochondria dysfunction and the downstream consequences in immune cells.

## Introduction

The impact of spaceflight on mitochondria has been known for decades, as reported in the muscle of rats (1), the ocular tissue of mice (2), and in human blood samples (3). In a comprehensive multi-omics analysis from human and animal samples flown in space, mitochondrial dysregulation was identified as a central hub across different species and organ tissues (4). Although mitochondria dysfunction can have profound consequences on human health, the underlying mechanisms of this spaceflight occurrence remain unresolved.

In this study, we investigated transcriptomic changes in blood samples collected from crewmembers during long-duration International Space Station (ISS) missions. Our intent is to identify potential signal transduction systems that regulate cell gene expression in response to the space environment, particularly the mechanisms underlying mitochondria dysfunction. Previously published gene expression data from astronauts’ blood samples showed dysregulated genes involved in DNA repair, oxidative stress, and protein folding/degradation in space (5). The NASA twin study revealed altered gene expression patterns related to the metabolic process of ROS, mitochondrial transport, hypoxia, and apoptotic mitochondrial changes (3). Other published studies include a transcriptomics analysis of ISS crewmembers’ leukocytes (6). A recent study of Inspiration 4 crewmembers’ blood using single cell gene expression technology also identified a set of “spaceflight genes” (7).

As our transcriptomics data was collected from the human blood samples in space, we also explored the mechanisms underlying other reported findings in astronauts’ blood. One of these findings is dysregulation of the immune system (8, 9). Studies conducted with astronaut biological specimens, cell culture systems, and animal models demonstrated that the immune system is affected during flight, and immune dysregulation, in part, persists after short- as well as after long-duration missions (10). Transcriptomic analyses of human peripheral blood mononuclear cells (PBMCs), splenocytes and purified T cells have demonstrated that the absence of gravity profoundly inhibits the capacity of immune cells to respond to *in vivo* (11) and *ex vivo* stimulations (12). Evidence of enhanced virulence of pathogens (13) and increased viral shedding indicate potential risks for astronauts´ health during long-duration missions (14). Recent studies using single cell analysis revealed simulated microgravity can induce inflammation and age like inflammatory changes in PBMCs (15). Additionally, it has been shown that simulated microgravity impairs bioactive lipids in monocytes, promoting immune dysfunction (16). Altered gene expression changes in primary and secondary lymphoid organs has also been reported in mice exposed to simulated microgravity (17). These findings demonstrate the complexity of immune dysfunction that arise during space travel. However, despite decades of research, the molecular mechanisms behind space environment-associated immune dysregulation are not fully understood.

The other reported finding is telomere lengthening. In the NASA Twin study, the average telomere length was shown to be greater in blood cells collected in space when compared to the ground controls (3). The length shortened rapidly after the missions (18). A mutational analysis conducted on *C elegans* during a short duration spaceflight mission also revealed telomere elongation (19). Likewise, a combination of simulated microgravity conditions and radiation exposure on myogenic cells showed telomere elongation in mice (20). While these studies highlight space induced telomere elongation across different species, the process in which it occurs is yet to be uncovered.

## Methods

### Sample collection

The data reported in this paper was collected from eleven astronauts. Four of the crewmembers were females. For each crewmember, blood was collected at two time points pre-flight, which varied from 240 to 85 days before launch for the first blood draw and from 125 to 23 days for the second blood draw. Onboard the ISS, blood was collected between 135 and 210 days after launch. In order to minimize any time-related effects, blood was drawn immediately prior to departing the ISS.

Blood was collected into an 8.5 mL BD ACD Vacutainer (BD Biosciences, Franklin Lakes, New Jersey) containing acid citric dextrose (ACD) solution. Blood tubes were then returned to the ground and transported at ambient temperature to Johnson Space Center (JSC) in Houston, Texas. The samples were received at JSC approximately 36 hours after blood draw on the ISS and then processed. The two preflight samples were collected at JSC and left unprocessed at ambient temperature for 36 hours in order to match the blood tube returning time from the ISS. At JSC, blood was diluted with phosphate buffered saline, and peripheral blood mononuclear cells (PBMC) were isolated using density gradient centrifugation. The PBMCs were then washed twice with PBS and lysed using Qiagen RLT Plus Buffer (Hilden, Germany). The lysates were frozen at -80°C until batch RNA isolation was performed. The results presented here correspond to gene expression of PBMC collected in space in comparison to the combined pre-flight time points.

### RNA isolation

Total RNA was isolated using a Qiagen AllPrep DNA/RNA/miRNA kit (Hilden, Germany) according to the manufacturer’s instructions. Briefly, the cells were homogenized using a QIAshredder (Hilden, Germany), passed through a DNA extraction column, precipitated with ethanol, passed through an RNA extraction column, treated with DNase, washed and eluted in nuclease free water. The concentration and purity of the RNA samples was determined using a Nanodrop 2000 instrument. The RNA isolates were frozen at -80°C until being shipped on dry ice for RNA-seq analysis. Although the blood samples were collected at different times for the 11 crewmembers, the RNA was isolated from PBMC in 3 batches. The batch effects were corrected in the data analysis. Isolated RNA from PBMC of the crewmembers yielded similar RNA concentrations between the blood samples collected in space and on the ground. The RNA integrity numbers (RIN) ranged from 7.2 and 9.3 for all samples, collected except for a pre-flight sample collected from Crew3 which was excluded from the analysis.

### RNA sequencing

Purified total RNA from all samples was submitted to the University of Wisconsin-Biotechnology Center for RNA QC, library construction (Illumina TruSeq) and NGS sequencing. Total RNA was assayed for purity and integrity via the NanoDrop One Spectrophotometer and Agilent 2100 Bioanalyzer, respectively. RNA libraries were prepared from samples that met the TruSeq^®^ Stranded Total RNA Sample Preparation Guide (15031048 E) input guidelines using the Illumina^®^ TruSeq^®^ Stranded Total (Globin) RNA Sample Preparation kit (Illumina Inc., San Diego, California, USA). For each library preparation, cytoplasmic ribosomal and globin RNA was removed using biotinylated target-specific oligos combined with paramagnetic beads tagged with streptavidin. Following purification, the reduced RNA was fragmented using divalent cations under elevated temperature. Fragmented RNA was copied into first stranded cDNA using SuperScript II Reverse Transcriptase (Invitrogen, Carlsbad, California, USA) and random primers. Second strand cDNA was synthesized using a modified dNTP mix (dTTP replaced with dUTP), DNA Polymerase I, and RNase H. Double-stranded cDNA was cleaned up with AMPure XP Beads (1X) (Agencourt, Beckman Coulter).

The cDNA products were incubated with Klenow DNA Polymerase to add a single ‘A’ nucleotide to the 3’ end of the blunt DNA fragments. Unique dual indexes (UDI) were ligated to the DNA fragments and cleaned up with two rounds of AMPure XP beads (0.8X). Adapter ligated DNA was amplified by PCR and cleaned up with AMPure XP beads (0.8X). Final libraries were assessed for size and quantity using an Agilent DNA1000 chip and Qubit^®^ dsDNA HS Assay Kit (Invitrogen, Carlsbad, California, USA), respectively. Libraries were standardized to 2nM. 2x150 paired-end sequencing was performed, using standard SBS chemistry on an Illumina NovaSeq 6000 sequencer and processed with bcl2fastq2 concersion software v2.20.

### Bioinformatics analysis

Bioinformatic analysis of transcriptomics data adheres to recommended ENCODE guidelines and best practices for RNA-Seq. Alignment of adapter-trimmed (21) 2x150 (paired-end; PE) bp strand-specific Illumina reads to the *Homo sapiens* GRCh38.p10 genome (assembly accession GCA_000001405.25) was achieved with the Spliced Transcripts Alignment to a Reference (STAR v2.5.3a) software (22), a splice-junction aware aligner, using Ensembl version 93 annotation. Expression estimation was performed with RSEM v1.3.0 (23).

Thirty-two samples from 11 crew members were assigned into pre-flight (n=21, with one crew member having only 1 pre-flight sample) and in-flight (n=11) groups based on the time of sample collection. Genes with low reads counts were removed using filterByExpr function within the edgeR package (24, 25) by applying default parameters. Gene counts were subsequently normalized with TMM algorithm using edgeR library. Differential gene expression analysis was performed using limma-voom pipeline (26). The design of the linear model for each gene was implemented using the model.matrix function in limma, including two terms, time (i.e., pre-flight, in-flight) and crew member ID. Incorporating crew member IDs as covariates in the model compensated for the individual differences of baseline gene expressions and increased the statistical power to identify changes.

To perform principal component analysis (PCA), values of counts per million (CPM) were calculated and log2-transformed. Batch effects due to different sample processing time were corrected using removeBatchEffect function from limma package. PCA was performed using the prcomp function from base package of R.

The DEGs were determined by a threshold of false discovery rate (FDR<0.01). These dysregulated genes were analyzed using Ingenuity Pathway Analysis (IPA, Qiagen, (27)) for pathways, functions and other biological consequences. IPA determines the statistical significance (*p*-value from Fisher’s exact test) based on the number of overlapping genes between the DEG in the present study and those in the IPA database for the specific pathways or functions. Whether a pathway is activated or inhibited is measured by the z-score, which is determined by the gene expression fold changes against the expected directions. The genes in the IPA database are compiled from comprehensive surveys of the scientific literature.

## Results

### Space environment-induced gene dysregulation

**Figure 1:**
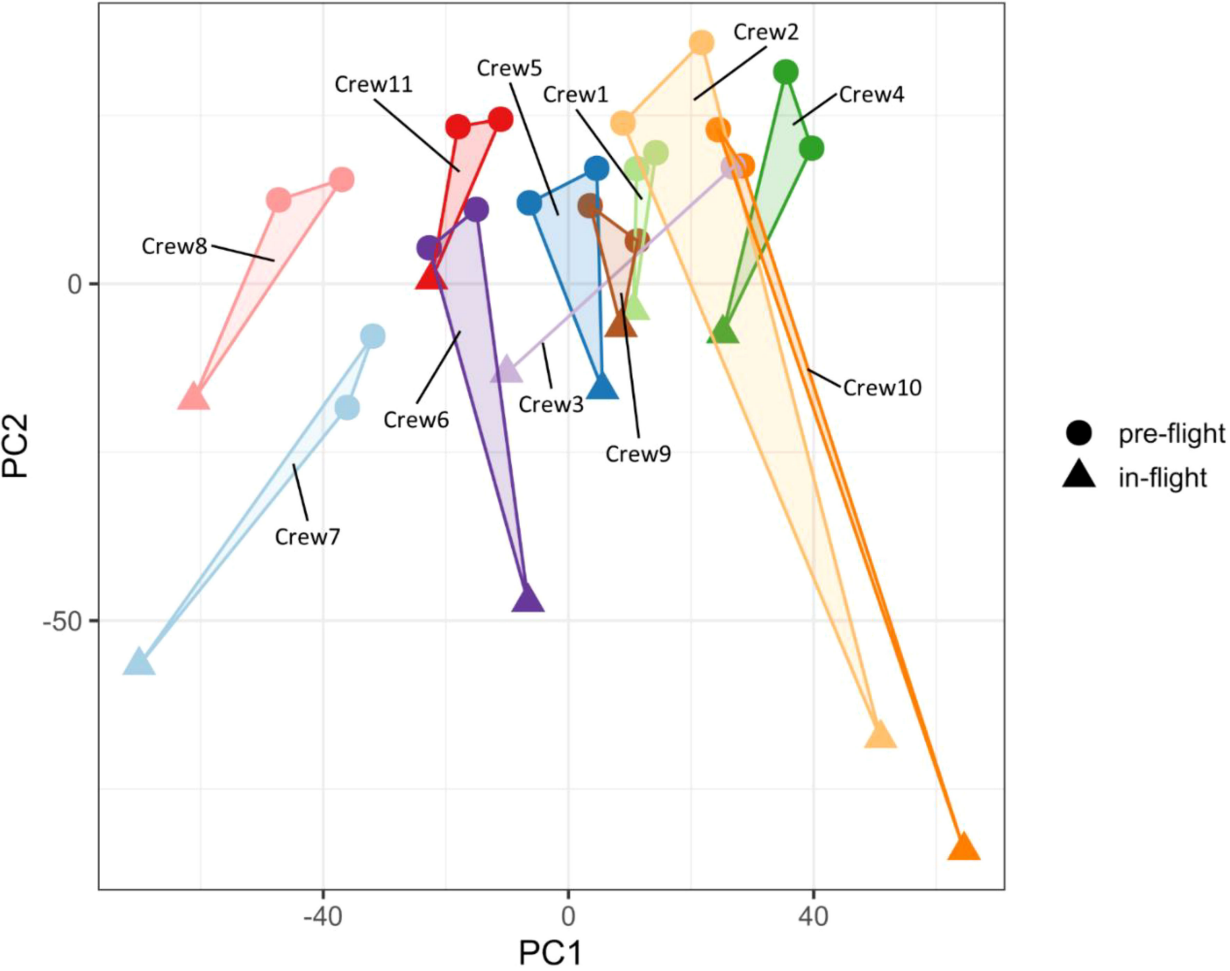
Principal component analysis of gene expression profiles. The in-flight PBMC samples were separated from pre-flight samples for all crew members.

Unsupervised principal component analysis (PCA) suggests changes in gene expression profiles occurred in PBMCs collected in-flight when compared with the pre-flight samples (**Figure 1**). The directions of the in-flight samples, which deviated from the pre-flight samples were consistent in all crew members (downwards along the PC2 axis), suggesting a similar pattern of gene expression changes in response to the space environment. The degree of changes varied across individual crew members, as demonstrated by the distances between in-flight and pre-flight samples, indicating the individual variations in the adaptation to the space environment.

With a threshold of false discovery rate (FDR) at 0.01, 1012 genes were upregulated and 1018 downregulated in the in-flight PBMCs when compared to the pre-flight samples. The top 100 DEG sorted by the FDR with the fold change values (FC) are listed in **Table 1**. The corresponding Volcano plot is shown in **Supplementary Figure S1**.

**Table 1:** Differentially expressed genes (DEGs) in PBMC of ISS crewmembers in space in comparison to pre-flight samples.

Symbol	FC	FDR	Symbol	FC	FDR
SEC14L2	2.8	4.7E-08	MAP1S	-3.5	4.4E-05
GPR174	1.8	5.8E-07	TMEM189	-1.8	4.5E-05
RUBCNL	4.0	1.3E-06	PLEKHG1	2.1	4.5E-05
ARID5B	1.9	2.3E-06	GPR15	2.0	4.5E-05
TMEM45B	2.0	8.1E-06	FAM78A	-1.6	4.7E-05
DNASE1L1	-1.6	1.3E-05	CD68	-1.6	4.8E-05
EOMES	2.2	1.3E-05	S100A10	-1.7	5.9E-05
CTSH	-1.7	1.3E-05	PRDM4	1.4	6.0E-05
PLEC	-1.9	1.8E-05	LILRB1	-1.8	6.0E-05
CDKN1A	-3.0	1.8E-05	TSPAN14	-1.8	6.0E-05
JDP2	-2.0	1.9E-05	TAGAP	1.5	6.0E-05
PPIF	-2.1	2.1E-05	HCP5	2.0	6.3E-05
FLVCR2	-2.8	2.5E-05	ZC3H12D	1.5	6.6E-05
PFN1	-1.6	2.5E-05	ARHGAP25	2.4	6.6E-05
CDK5R1	-2.2	2.5E-05	VIM	-1.6	6.6E-05
GPR65	1.7	2.5E-05	SH3TC1	-1.6	6.6E-05
CYC1	-1.7	2.5E-05	ACTG1	-1.3	6.7E-05
VEGFA	-4.5	2.5E-05	GRASP	-3.1	6.7E-05
LRIG1	1.8	2.6E-05	HK1	-1.3	6.7E-05
ARL5B	1.5	2.6E-05	ID2	-2.8	6.7E-05
CST3	-1.6	2.7E-05	MAFF	-1.8	6.7E-05
RUNX1	-1.9	3.1E-05	ZDBF2	1.7	6.8E-05
RASGRP1	1.8	3.1E-05	EPM2AIP1	1.3	6.8E-05
SLAMF1	1.7	3.1E-05	UBAC1	-1.4	7.0E-05
NABP1	2.9	3.4E-05	CBLB	1.5	7.1E-05
TNFRSF13C	1.9	3.4E-05	LGALS3	-2.0	7.3E-05
SCML1	-1.8	3.4E-05	NTSR1	-2.9	7.4E-05
TTC7A	-1.7	3.4E-05	CD302	-1.5	7.4E-05
DDIT4	-1.6	3.4E-05	TNFRSF21	-2.8	7.8E-05
NAPSB	-1.7	3.4E-05	CTSB	-1.4	8.0E-05
GLS	1.4	3.5E-05	SERPINF1	-2.1	8.1E-05
BCAT1	-1.7	3.5E-05	PNRC1	1.4	8.4E-05
CXCL8	4.4	3.5E-05	TOPORS	1.6	8.4E-05
MRPL23	-1.5	3.5E-05	CYP2S1	-2.5	8.4E-05
PIM2	2.0	3.5E-05	KLF2	1.7	8.5E-05
UPP1	-2.3	3.5E-05	ARHGAP15	1.4	8.6E-05
IRF5	-1.7	3.5E-05	NDFIP1	-1.5	8.6E-05
EFNA1	-6.6	3.5E-05	TGFBI	-1.7	8.6E-05
NFKBID	1.9	3.5E-05	CAMSAP1	-1.9	8.8E-05
IRF4	-1.9	3.5E-05	TRIM39	1.6	8.9E-05
ALDH2	-1.5	4.0E-05	PMP22	-3.6	9.6E-05
MAN2B1	-1.4	4.0E-05	PTGS2	2.8	9.6E-05
RNH1	-1.7	4.0E-05	CHN1	2.1	9.9E-05
FAM84B	1.5	4.0E-05	TUBD1	1.8	1.0E-04
SH2D1A	1.9	4.0E-05	P4HB	-1.3	1.0E-04
LILRB4	-1.9	4.0E-05	C1orf132	2.3	1.0E-04
GSTP1	-1.7	4.2E-05	SEC13	-1.3	1.0E-04
CD28	1.6	4.2E-05	ADORA2A-AS1	4.7	1.0E-04
ASB6	-1.5	4.3E-05	ABHD12	-1.7	1.0E-04
RGS2	2.0	4.3E-05	MTMR11	-1.5	1.0E-04

The top 100 DEGs sorted by FDR are presented. FC.

### Cellular pathways and gene functions affected by the space environment

**Figure 2:**
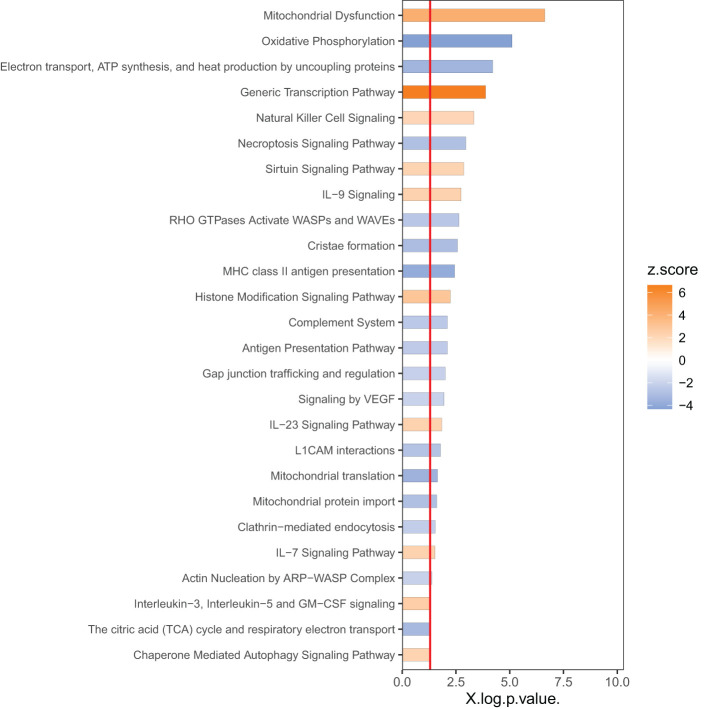
Top 50 canonical pathways identified by IPA.

In order to identify the responsive canonical pathways in the space environment, we applied Ingenuity Pathway Analysis (IPA) with a threshold of p<0.05 as the cutoff. In IPA, the cutoff of p<0.05 indicates statistical significance of change based on the overlapping DEGs associated with a specific pathway, whereas the z-score indicates significance in activations or inhibitions of these pathways (See Methods). **Figure 2** shows the top pathways with a |z| -score threshold of 2. The pathways impacted by spaceflight are mostly involved in i) metabolism and mitochondria dysfunction, including oxidative phosphorylation and tricarboxylic acid cycle (TCA), ii) immune responses, such as natural killer cell signaling, interleukin signaling and hypoxia-inducible factor 1-alpha signaling (HIF-1α), iii) actin nucleation and iv) cell death/survival including sirtuin signaling, necroptosis and autophagy.

**Figure 3:**
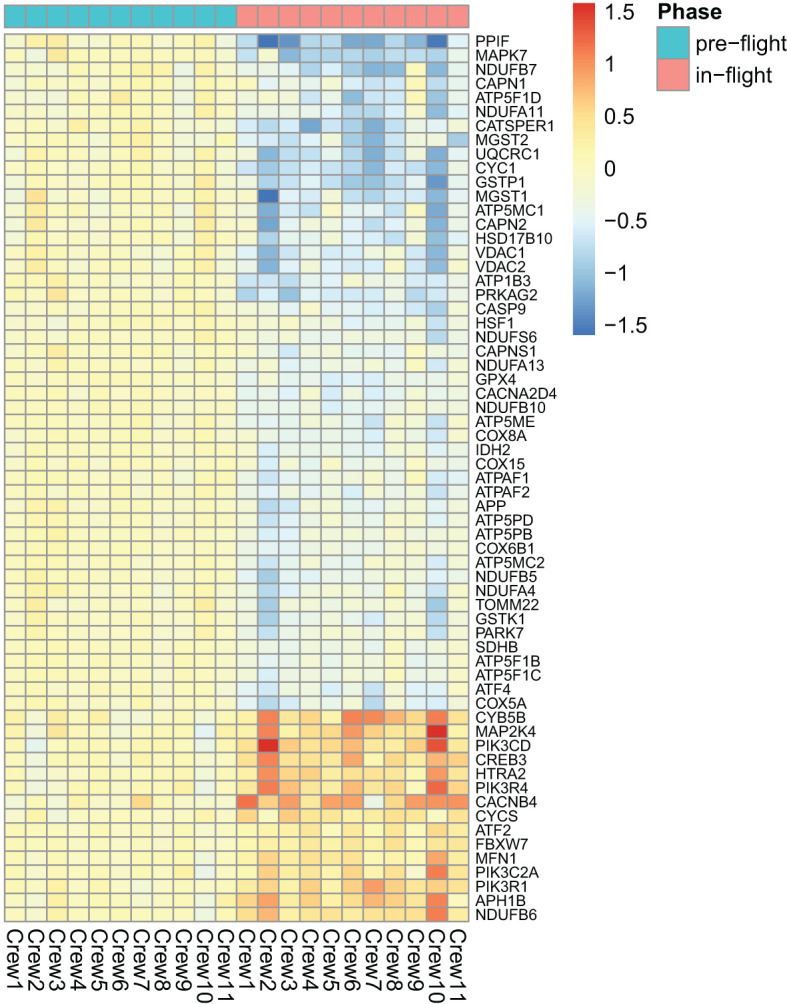
Heatmap of the dysregulated genes involved mitochondria dysfunction in space.

As shown in **Figure 2**, among the top dysregulated pathways are mitochondria dysfunction and oxidative phosphorylation. These pathways are dysregulated due to expression of the number of the genes in the mitochondria as shown in **Figure 3**. Several pathways related to mitochondria/ATP production have also been identified including cristae formation and TCA cycle. Among mitochondrial genes, OXPHOS is shown to be impaired at multiple complex enzymes. Dysregulated complex I genes include: NDUFB7(-1.6, 0.004), NDUFA11(-1.4, 0.002), NDUFS6(-1.3, 0.006), NDUFA13(-1.2, 0.005) NDUFB10(-1.3,<0.001), NDUFB5(-1.4,<0.001), NDUFA4(-1.3, 0.01) and NDUFB6 (1.4, 0.002). Most notably, complex I functions to oxidize NADH and pump protons. Dysregulated complex III genes include: UQCRC1 (-1.6, <0.001) and CYC1 (-1.7, <0.001). Complex III contributes to the transfer of electrons from ubiquinol to cytochrome c. Dysregulated genes in complex IV include: CYC(1.3, 0.005), COX8A(-1.3, 0.002), COX15(-1.2, 0.007), and COX6B1 (-1.3, 0.001). Complex IV serves as the last step of OXPHOS and generates water by transferring electron from cytochrome C to O2. Dysregulated Complex V genes include: ATP5F1B(-1.2,0.005), ATP5F1C(-1.2,0.006), ATP5F1D(-1.4, 0.008), ATP5MC1(-1.5, 0.007), ATP5MC2(-1.3, 0.003), ATP5ME(-1.3, 0.003), ATP5PB(-1.2, 0.003), ATP5PD (-1.3, 0.002). ATP synthase, or complex V, is vital in the conversion of ADP to ATP. All dysregulated genes are encoded by nDNA and no mtDNA genes were shown dysregulated on the heat map. DEGs of these enzyme subunits demonstrates impaired ATP production and cellular metabolism.

One of the dysregulated pathways that is not in **Figure 2** is G-protein coupled receptor (GPCR) signaling, which has p=0.001 and z=1.3. **Table 2** shows the list of dysregulated GPCRs. The reason for a lower z-score is that some of the receptors are upregulated, but some downregulated. Besides adhesion GPCRs, dysregulated GPCRs are organized by calcium flux modulation. Receptors coupled to G alpha q activate the IP3 calcium pathway through the alpha subunit. Receptors coupled to G alpha S activate adenylate cyclace which in turn activates pKA to open calcium channels RyR calcium channels on the endoplasmic reticulum. Receptors coupled to G alpha i/o proteins activate the PLC gamma pathway through the beta-gamma subunit. Despite being coupled to different G proteins these receptors maintain the ability to increase intracellular calcium, as discussed below.

**Table 2:** Dysregulated GPCR in space.

Symbol	Entrez Gene Name	Fold change	FDR
	Adhesion GPCRs		
ADGRG3	adhesion G protein-coupled receptor G3	3.2	1.2E-03
ADGRE3	adhesion G protein-coupled receptor E3	2.7	5.2E-04
ADGRA2	adhesion G protein-coupled receptor A2	-1.6	1.9E-03
ADGRE1	adhesion G protein-coupled receptor E1	-1.3	1.2E-03
	Upregulated GPCRs that increase cytosolic Ca2+		
HCAR2	hydroxycarboxylic acid receptor 2	5.5	2.8E-04
CXCR2	C-X-C motif chemokine receptor 2	4.9	6.0E-03
HCAR3	hydroxycarboxylic acid receptor 3	4.1	3.7E-04
FPR2	formyl peptide receptor 2	3.5	1.5E-03
FFAR2	free fatty acid receptor 2	3.0	1.9E-03
C5AR2	complement C5a receptor 2	2.6	3.8E-03
CCR3	C-C motif chemokine receptor 3	2.4	7.2E-03
ADORA2A	adenosine A2a receptor	2.2	2.1E-03
CCR1	C-C motif chemokine receptor 1	2.2	7.5E-03
PTAFR	platelet activating factor receptor	2.2	2.1E-03
FPR1	formyl peptide receptor 1	2.1	2.1E-03
P2RY13	purinergic receptor P2Y13	2.1	6.9E-03
GPR15	G protein-coupled receptor 15	2.0	4.5E-05
GPR174	G protein-coupled receptor 174	1.8	5.8E-07
GPR65	G protein-coupled receptor 65	1.7	2.5E-05
GPR18	G protein-coupled receptor 18	1.5	1.7E-03
P2RY10	P2Y receptor family member 10	1.5	2.6E-04
S1PR5	sphingosine-1-phosphate receptor 5	1.5	6.3E-04
	Downregulated GPCRS that increase cytosolic Ca2+		
S1PR2	sphingosine-1-phosphate receptor 2	-3.1	2.5E-03
NTSR1	neurotensin receptor 1	-2.9	7.4E-05
P2RY2	purinergic receptor P2Y2	-2.7	2.6E-03
GPRC5C	G protein-coupled receptor class C group 5 member C	-2.3	1.2E-03
PTGIR	prostaglandin I2 receptor	-1.7	1.1E-03
VIPR1	vasoactive intestinal peptide receptor 1	-1.7	5.1E-03
P2RY1	purinergic receptor P2Y1	-1.6	2.4E-03
PTGDR2	prostaglandin D2 receptor 2	-1.6	7.7E-03
	Downregulated GPCRS that decrease cytosolic Ca2+		
GPR35	G protein-coupled receptor 35	-2.0	1.8E-03
GABBR1	gamma-aminobutyric acid type B receptor subunit 1	-1.7	5.5E-03
	Upregulated GPCRs that decrease cytosolic Ca2+		
SSTR3	somatostatin receptor 3	1.4	9.2E-03
	Unconventional GPCRs with little known Ca2+		
ACKR3	atypical chemokine receptor 3	-8.5	1.0E-03

When analyzing diseases and gene function annotations, IPA (|z| score threshold of 2) identified downregulated functions including endocytosis, cell migration, cell-cell contact and cell maturation (**Table 3**). All of the top diseases and functions have small *p*-values.

**Table 3:** Diseases or functions that are associated with the DEGs as identified by IPA.

Diseases or Functions Annotation	Activation z-score	p-value
Endocytosis	-3.2	1.2E-19
Endocytosis by eukaryotic cells	-3.0	8.1E-16
Engulfment of cells	-3.0	1.8E-16
Infection of cells	-2.8	1.1E-16
Cell movement of antigen presenting cells	-2.7	1.1E-10
Engulfment of myeloid cells	-2.6	1.2E-12
Infection by RNA virus	-2.6	4.0E-30
Phagocytosis of myeloid cells	-2.6	6.5E-13
Phagocytosis of blood cells	-2.5	2.2E-14
Cell movement of macrophages	-2.5	3.5E-09
Phagocytosis of antigen presenting cells	-2.4	2.5E-08
Viral Infection	-2.4	7.0E-37
Internalization of cells	-2.4	1.1E-13
Cell-cell contact	-2.3	5.6E-07
Infiltration by T lymphocytes	-2.3	2.5E-09
Phagocytosis by macrophages	-2.3	4.2E-08
Phagocytosis	-2.3	1.0E-15
Engulfment of blood cells	-2.2	3.2E-14
Phagocytosis of phagocytes	-2.2	2.6E-09
T cell migration	-2.2	5.3E-18
Cellular infiltration by macrophages	-2.2	1.4E-07
Systemic autoimmune syndrome	-2.2	2.2E-25
Development of progenitor cells	-2.2	5.3E-15
Leukocyte migration	-2.2	7.8E-24
Cell movement of blood cells	-2.1	6.1E-24
Immune response of myeloid cells	-2.0	2.2E-14

The DEGs were also analyzed using the GSEA method. The results of cellular components and molecular functions are shown in **Supplementary Table S2**.

## Discussion

Mitochondrial dysfunction has been a documented consequence of space flight in both humans and animals (1–3). Understanding the underlying mechanisms and implications of mitochondrial dysfunction will provide crucial knowledge for future spaceflight missions. This discussion will dive deeper into the molecular and cellular aspects that are found within the 2030 differentially expressed genes and results generated from IPA and its implications in spaceflight.

### GPCRs

G protein Coupled Receptors (GPCRs) are the largest class of membrane receptors that are abundantly expressed in various cell types including T lymphocytes (28). Our study demonstrates a dysregulation in GPCR related genes (**Table 2**). GPCRs function by converting extracellular signals into biochemical responses that can activate or suppress cell function. Activation of GPCRs can occur through a ligand dependent and independent manner, activating a signaling cascade that’s both receptor and cell specific. Ligand dependent GPCR activation requires a ligand to bind to the receptor while ligand independent GPCR activation can occur via mechanical force or membrane modifications that induces a conformational change (29). Our data shows the upregulation of 18 ligand dependent GPCRs that have been linked to increasing cytosolic calcium and promoting calcium mobilization (**Table 2**). Activated GPCRs undergo a conformational change that exchanges GDP for GTP, causing the dissociation of Gα and βγ subunits (30). Depending on their subtype, the subunits can modulate cytosolic calcium concentration and mobilization through the activation of PLC-beta and cAMP signaling cascades (31). Our dataset shows the downregulation of GABBR1 and GPR35 (**Table 2**) which have been implicated in decreasing cytosolic calcium levels, thereby elevating intracellular calcium (32). Adhesion GPCRs that were dysregulated include, ADGRA2, ADGRE1, ADGRE3 and ADGRG3 (**Table 2**) and are proposed mechanoreceptors due to their large NH2-terminal region (33). Dysregulation of mechanosensing GPCRs may be attributed to microgravity during spaceflight. Various studies on plants, microbes, yeasts and mammal cells have shown that long exposure of microgravity exhibits changes in mechanotranduction and serving as an adaptive response to a decreased pressure environment (34).

Furthermore, there was dysregulation in genes that modulate GPCR activity, providing a mechanism to enhance and prolong calcium influx. GNG2 (FC=1.4, FDR=0.009) was upregulated in our data and encodes for the G-protein gamma 2, a subunit on the βγ complex. A study isolating G-protein γ subunit has been shown to enhance the lipase activity of the GαqGTP–PLCβ complex, promoting more cytosolic calcium (35). Our transcriptomic data also shows the down regulation of PLPP3 (-6.5, 0.009), RGS1 (-2.2, 0.001), and RGS16 (-5.9, 0.002) which are negative regulators of G protein signaling. An upregulation of 18 GPCRs that increase cytosolic calcium demonstrates a potential enhancement of calcium influx during spaceflight. This is further elucidated by the dysregulation of GPCR modulatory genes.

### Cell membrane

Lipids function to regulate the fluidity, and structure of the cell membrane and regulate the location of membrane bound receptors (36). The integrity and composition of the lipid membrane has been shown to modulate GPCRs. A study conducted on β2-adrenergic receptors demonstrated that lipid head groups could allosterically modulate ligand binding and GPCR activation (37). This study also demonstrates that anionic lipids were able to enhance the open conformation of β2-adrenergic receptors compared to other lipids. Another study showed that increasing membrane fluidity led to a more active confirmation on GPCRs (38). The lipid membrane is comprised of phospholipids, glycolipids, sphingolipids and sterols. Our data shows the downregulation of sphingolipid metabolism (**Figure 2**). Impaired metabolism of sphingolipids was shown to enhance the activity of serotonin 1A receptors (39). Additional dysregulated genes involved in the maintenance of the lipid membrane include: SEC14L2 (2.8, <0.001), PLA2G15 (-1.5, 0.005), LIPA (-1.6, 0.001), NPC1 (1.4, 0.006), NPC2 (-1.4, 0.001), STARD4 (-1.5, 0.001), AUP1 (-1.3, 0.001), HILPDA (3.4, <0.001), APOL2 (1.7, 0.001), APOL3 (1.3, 0.008), APOL6 (1.3, 0.002), DGAT2 (3.8, 0.001), SCD (-2.3, 0.002) and PEMT (-1.6, 0.005). Cholesterol has also been shown to modulate stability, signaling and ligand affinity of multiple GPCRs (40). Simulated microgravity was found to dysregulate the metabolism of sterols, phospholipids, sphingolipids, glycerolipids, and others in epidermal stem cells (41). Thus, we propose that microgravity may modulate both mechanoreceptor and ligand GPCRs by dysregulating the surrounding lipid environment.

It has been suggested that cells respond to microgravity through cytoskeleton reorganization (42). Interestingly, activation of GPCRs induce cytoskeleton reorganization and may suggest a potential crosstalk between microgravity and GPCRs. Moreover, simulated and real microgravity altered the organization of actin filaments and microtubules (43). GPCRs can initiate a Rac1-WASP family signaling cascade that activates the ARP2/3 complex to induce actin polymerization (44). Rac1 can be inhibited by specific GTPase activating proteins (GAPs) and therefore its activity is not solely tied to the activation of GPCRs. Our data shows the upregulation of rac1 GAP proteins: CHN1 (2.1, >0.001), ARHGAP25 (2.4, <0.001), and ARHGAP15 (1.4, 0.001), and downregulation of RAC1 (-1.4, 0.004), which may suggest that the GPCR cytoskeleton remodeling cascade is disrupted by GTPase activating proteins rather than the lack of an initial GPCR signal. A study done on human MG-63 osteoblast-like cells revealed that silencing rac1 suppressed microgravity induced alterations of the cytoskeleton (45). Therefore, the downregulation of the rac1 pathway could also be a potential compensatory mechanism for the impaired cytoskeleton from microgravity. In the present study, the actin nucleation ARP-WASP complex pathway (**Figure 2**) was also down regulated. The associated genes include downregulation of actin (ARPC3 (-1.3, 0.001), ARPC4 (-1.2, 0.003) and ARPC1B (-1.4, 0.001)) and integral proteins (ITGAM (-1.3, 0.003) and ITGB2 (-1.3, 0.001)). Other dysregulated actin cytoskeleton related genes include PLEC (-1.9, <0.001), PSTPIP2 (-1.6, <0.001), ACTB (-1.4, 0.001), ACTG1 (-1.3, <0.001), MYH11 (16, 0.001), MYL6B (-1.6, 0.003), and MYO10 (-2.4, 0.002). The disruption of the actin cytoskeleton has also been shown to enhance ligand binding of serotonin 1a receptors by either increasing the probability of GPCRs interacting with G proteins or changing conformational dynamics (46). Another study using serotonin 1a receptors showed that destabilizing the actin cytoskeleton enhanced the GPCRs signaling efficiency (47). Thus, cytoskeleton dysregulation induced by microgravity may modulate GPCRs and contribute to increased levels of cytosolic calcium.

### Mitochondria dysfunction

Our data revealed mitochondrial dysfunction, and downregulation of oxidative phosphorylation (OXPHOS) and tricarboxylic acid cycle (TCA cycle) (**Figure 2**), indicating a clear impact of the space environment on cellular respiration glucose metabolic and ATP production (**Figure 3**). Spaceflight has previously been shown to impact OXPHOS and induce mitochondrial dysfunction (3, 7). A comprehensive multi-comics analysis from human and animal samples in space also identified mitochondrial dysregulation as a central hub across different species and organ tissues (4). The impact of spaceflight on mitochondria has also been reported previously in the muscle of rats (1, 48) and in the NASA twin study (3). Prolonged exposure (~6 months) to space conditions reduced mtDNA and mtRNA production in astronauts’ hair follicles (49). The cell-free mitochondrial DNA concentration in circulating blood increased in astronauts and has been a suggested biomarker for stress or immune responses related to environmental space factors (50). In mice flown on the space shuttle, it was also reported that spaceflight induced mitochondrial oxidative damage in ocular tissue (2). Our data shows the upregulation of MICU3 (1.9, 0.008), a gene that regulates calcium uptake into the mitochondria. Using mice, it was demonstrated that over-expression of MICU3 increased mitochondrial calcium levels while MICU3 knockout mice had reduced levels of mitochondrial calcium (51). Transcriptomic data also points to increased intracellular calcium via up regulation of STIM2 (1.3, <0.001). STIM proteins trigger the influx of calcium into the ER through a plasma membrane bound calcium channel when it detects low levels of ER stored calcium (52). Downregulation of STIM2 in neurons decreased mitochondrial calcium levels, calcium release from the ER, and improved mitochondrial function, demonstrating its role in mediating mitochondrial calcium concentrations (53). Our data also shows the upregulation of ITPRIPL1, PLCL1, PLCD1 genes, indicating modulated IP3 activity. Likewise, our data shows the downregulation of CACNA2D4, which has been shown to lower the voltage required for the calcium channel to open (54).

Calcium overload disrupts the mitochondria by destabilizing cristae, impairing oxidative phosphorylation, and inducing the opening of the mitochondrial permeability transition pore (mPTP) complex (55). A dysregulation in the cristae network was noted in calcium overloaded mitochondria and aligns with the downregulation of cristae formation from our dataset (**Figure 2**). This phenomenon may occur due to the formation of calcium phosphate precipitates that destabilize the cristae structure (56). Likewise, it has been reported that high mitochondrial calcium levels impair the rate of ADP phosphorylation in OXPHOS (55). This is consistent with our data as oxidative phosphorylation was among the top 3 down regulated pathways (**Figure 2**). Calcium regulates the mPTP which is a non-selective pore. This complex is triggered by calcium concentration which induces an open confirmation that is either transient or sustained (57). A transient open confirmation allows for the efflux of Ca2+ and ROS and serves a regulatory function. During calcium overload, a sustained open confirmation allows for ion and solute influx that induces apoptosis and necroptosis by releasing pro-apoptotic proteins (58).

In addition, spaceflight is known to alter the general metabolic state of living organisms (59). Microgravity has been shown to associate with a decrease in key glycolysis enzymes in hematopoietic progenitor cells (60). In muscle biopsies taken from astronauts after short (~11 days in space) and long (~180 days in space) duration missions, proteins and genes involved in TCA and fatty acid beta-oxidation decreased (61). Moreover, the TCA activity and the glycolysis/gluconeogenesis ratio was reduced in mouse muscles during spaceflight (62). However, simulated microgravity and real space conditions seems to promote or inhibit glycolysis or TCA, depending on the cell type and *in vitro*/*in vivo* conditions (63). In a study of mice flown in space for different durations (64), it was reported that some mitochondria related genes were also dysregulated, depending on the flight duration. As the flight duration is proportional to the dose of space radiation exposure that the mice received, the contribution of radiation to mitochondria dysfunction cannot be ruled out. It is likely that some of the biological effects of spaceflight are caused synergistically by microgravity, cosmic radiation and other stress factors.

### Endocytosis, cell migration and cell adhesion

In our study, endocytosis, phagocytosis and engulfment were identified amongst the highest down regulated cell processes (**Table 3**). The dysregulated genes associated with endocytosis are listed in **Supplementary Table S1**. Endocytosis regulates several functions including signal transduction, membrane composition, mitosis, synaptic vesicle recycling, adhesion, lipid homeostasis, motility and cell morphogenesis (65). Therefore, the downregulation of genes involved in cell migration and adhesion comes as no surprise. Endocytosis is a fundamental process in T cells due to their role in membrane receptor trafficking recycling and targeted degradation. Bystander T cell receptor (TCR) recycling is a clathrin mediated endocytosis dependent process which was down regulated in our dataset (**Table 3**) (66). More specifically our data shows the downregulation of AP2S1 (-1.5, <0.001), AP2M1 (-1.3, <0.001), DNM2 (-1.2, 0.003), and DNMT1 (-1.3, 0.009) indicating an impaired recruitment for clathrin coating and pit scission. Moreover, endocytosis regulates T cell receptor (TCR) signaling, antigen discovery by trogocytosis and activated cell growth (57). Additionally, endocytosis requires cell surface receptors for recognizing nutrients and pathogens (67). Mechanisms underlying endocytosis in microgravity conditions are not fully understood but macrophages have been shown to exhibit impaired phagocytosis, adhesion migration and cytokine production in space (68). Similarly, long-term cultures of dendritic cells in simulated microgravity resulted in reduced antigen uptake (69) and fungal conidia uptake by phagocytosis (70). Downregulation of phagocytic surface markers CD32 and CD64 were also seen in astronauts’ monocytes after 5-11 days in space (71). Endocytosis is a key component for cell fate determination and cell migration (72), which are down regulated processes in our dataset (**Supplementary Table S1**). It should also be noted that RUNX1 (-1.9, <0.001) and GRASP (-3.1, <0.001), regulators of cell adhesion, migration and endocytosis (73, 74), were among the top down regulated genes from our data. Moreover, it has been reported that after short duration space flight the surface expression of CD62L and HLA-DR, key factors in endothelial cell adhesion and tissue migration, were significantly reduced (75).

Modifications in the cytoskeleton are required for these processes to occur as actin polymerization drives the morphological changes that enable cells to undergo division, phagocytosis and migration. The actin cytoskeleton is crucial for the adaptive immune response due to its role in the organization and function of the immune synapse during antigen recognition (76). The formation of new actin filaments from actin monomers is regulated by the Arp2/3 complex among others (77). A reduction in cell movement and adhesion as seen in our analysis (**Table 3**) may be due to the impact of spaceflight on the actin cytoskeleton. As mentioned above, the Rac1-Arp2/3 pathway and actin nucleation ARP-WASP complex were down regulated (**Figure 2**). The Arp2/3 complex relies on ATP for its activation therefore impaired oxidative phosphorylation due to calcium overload may play a key role in its dysregulation.

### Cell survival

The mitochondrion is a multifaceted organelle, responsible for cellular ATP production but also modulates apoptosis, necroptosis, ferroptosis, pyroptosis and autophagy (78). In the present study, BCL6 (1.5, 0.004) was upregulated in space. Over expression of BCL6 has been shown to inhibit apoptosis in differentiation-induced myogenic cells (79). Our data also shows the downregulation of BCL7B (-1.8, <0.001). Knockdown of BCL7B in human gastric cancer cells were shown to suppress cell death (80). Furthermore, our data showed the downregulation of two calcium mediated apoptotic pathways that are independent of the mitochondria, providing a survival mechanism for cells during calcium overload. A dysregulation of calcium induces ER stress by impairing calnexin and calreticulin, which under normal conditions facilitates proper protein folding (81). Our data shows the downregulation of Calnexin (CANX (-1.3, <0.001)) and calreticulin (CALR (-1.3, 0.001)), suggesting that the unfolded protein response (UPR) system is activated. Activation of the UPR leads to a cascade that ultimately causes apoptosis by CHOP (82). Interestingly, our data shows the downregulation of associated UPR proteins ATF4 (-1.3, 0.001), and ATF3 (-1.5, 0.009), indicating impaired apoptosis from calcium induced ER stress. Other dysregulated calcium-dependent apoptosis genes in our data set were CAPN2 (-1.5, 0.002), CAPNS1 (-1.3, 0.002), CAPN1 (-1.3, 0.01), and CASP9 (-1.3, 0.006). Dysregulated genes that were found to promote cell survival include PIM2 (2.0, <0.001), PIK3CD (1.6, 0.001), PIK3R4 (1.5, 0.001), TMEM52B (3.4 0.003), MOAP1 (-1.4, 0.003), and TNFRSF10A (-1.3, 0.009).

Importantly, our data revealed the downregulation of genes involved in the intrinsic death pathway for T cells. Similarly, calcium overload in the mitochondria induces apoptosis through the same mechanism. The intrinsic death pathway in T cells can be activated by TCR activation, DNA damage, developmental cues, or growth factor deprivation (83). Likewise, an increased level of mitochondrial calcium can also initiate these events in other cell types. These signals trigger the opening of the mPTP in the mitochondria and release pro-apoptotic proteins: CytC, Endo G, AIF and others (84). CytC forms an apoptosome that activates CASP9 which leads to apoptosis via a caspase cascade (85). On the other hand, Endo G and AIF mediate apoptosis through a caspase independent pathway, translocating to the nucleus and degrading chromatin (86). Our data shows the downregulation of PPIF (-2.1, >0.001) and PACS2 (-1.4, 0.007), demonstrating an impaired release of apoptotic proteins from the mitochondria. PPIF knockdown mice showed impaired cytochrome C release, inhibition of necroptosis and protected autophagy (87), aligning with our data (**Figure 2**). Likewise, PACS2 depleted cells have been shown to inhibit apoptosis by blocking the release of cytochrome c (88). Additionally, our data showed the downregulation of CASP9 (-1.3, 0.006), CARD14 (-1.8, 0.004) and PYCARD (-1.5, <0.001), indicating impaired initiation and recruitment of caspases. Our analysis also showed the downregulation of AIF genes AIFM2 (-1.6, 0.007) and AIFM3 (-1.8, 0.01). IL-7 signaling (**Figure 2**) was also upregulated in our data, which most notably inhibits the intrinsic apoptotic pathway by up-regulating anti-apoptotic proteins (87). BIRC6 (1.2, 0.008), an inhibiting apoptosis protein (IAP), was also upregulated.

Avoiding cellular apoptosis is key for T cell survival, but cellular stress can activate senescence, rendering lymphocytes inactive. Autophagy has a complex relationship with senescence, but several studies show that autophagy can suppress senescence in cells with a dysfunctional mitochondrion (89). Furthermore, autophagy is known to play a role in maintenance of T cells (90) and promotes longevity of organisms. Our data indicated activation of chaperone mediated autophagy (CMA) (**Figure 2**). CMA has been linked to enhancing TCR activation by degrading inhibitory signals and increasing cell survival by degrading pro-apoptotic proteins (91). Additionally, autophagy was upregulated in our dataset as a result of dysregulation of ATG12 (1.2, 0.004), ATG2A (2.0, 0.003), ATG2B (1.4, 0.004) and other genes. Other autophagy related genes such as MAP1LC3A (2.7, <0.001) and RUBCNL (4.1, <0.001) (**Table 1**) were also upregulated. Likewise, TMEM189 (-1.8, <0.001) and PPP1CA (-1.4, <0.001), negative regulators for autophagy, were downregulated. Additionally, downregulation of SLC1A5 (-1.9, 0.001) and SLC7A5 (-3.4, <0.001) in our dataset suggests reduced amino acid transport and starvation of the cells. Moreover, a downregulation of endocytosis as shown in **Table 3** contributes to the nutrient starvation of cells which induces autophagy (92). The sirtuin signaling pathway was also found to suppress oxidative stress induced senescence in osteoporotic bone marrow mesenchymal stem cells (93). Our findings show the upregulation of the sirtuin signaling pathway (**Figure 2**). The suppression of senescence from the sirtuin pathway occurs by halting telomere attrition and upregulating DNA repair mechanisms. Interestingly mammalian cells contain 6 SIRT proteins, three of which (SIRT1,3,6) can function as gene expression suppressors, to stabilize chromatin (94).

Moreover, T cells must overcome telomere attrition to avoid senescence (95), An increased telomere length was reported in T cells during the NASA twin study (3). The increased telomere length was independent of telomerase, suggesting an aberrant pathway for telomere maintenance and lengthening. It has been proposed that the transient activation of telomerase-independent ALT pathway is a response to chronic oxidative damage and serves as a mechanism for cell survival (96). Our data shows the upregulation of 8 genes (NR2C2 (1.4, 0.005), CHD4 (1.5, <0.001), SMC4 (1.4, <0.001), RPA3 (1.3, 0.005), RPA2 (1.4, 0.008), ATRIP (1.4, 0.002), TOP3A (1.4, 0.007), and PCNA (1.6, <0.001)) that activate the ALT pathway, and the downregulation of 1 inhibitory gene (ERCC1 (-1.5, <0.001)) (97). These dysregulated genes may indicate an adaptive response to the chronic oxidative stress endured by T cells during spaceflight. In addition, downregulation of senescence biomarker genes was noted in our data and included CDKN1A (-3.0, <0.001), CDKN2AIP (-1.3, 0.004), TP53I13 (-1.6, <0.001), E2F4 (-1.3, <0.001), PPP1CA (-1.4, <0.001), and DEC1 (BHLHE40, -2.7, <0.001). Likewise, our data did not show expression changes to GLB1 which suggests unchanged sa-β-galactosidase activity. Altogether, the downregulation of apoptosis, necroptosis, and upregulation of chaperone mediated autophagy, sirtuin signaling and ALT pathway suggests a mechanism for T cell survival and telomere lengthening during spaceflight.

Emerging research demonstrates that telomeric RNA (TERRA) increases with chronic low dose radiation exposure (98). Importantly, TERRA is a mediator for ALT. TERRA was increased in the blood of high-altitude climbers and *in vitro* simulated radiation studies, but not in simulated microgravity. However, the simulated microgravity experiment did not investigate the synergistic effects microgravity may have with space radiation nor the effects of long-term microgravity exposure. PBMCs from the experiment were only exposed to simulated microgravity for 25 hours, which may not have provided sufficient time for key adaptational changes that link microgravity to telomere elongation. TERRA is released as a cellular response to telomeric damage from ROS, hypoxia, DNA breaks and other stressful signals. While space radiation plays a role in these stress responses, microgravity has been shown to induce similar molecular stressors (99). Levels of telomeric RNA increase in a radiation dose dependent manner (100, 101), suggesting that the duration of stress exposure influences telomere elongation and therefore should also be considered in the context of long term microgravity exposure.

### Compromised immune function

Impaired immune function has been documented in astronauts during spaceflight and after returning to earth (102). T cell activation initiates a phosphorylation cascade that leads to gene expression changes that modulate effector functions (103). Protein tyrosine kinases serve as the phosphorylating enzymes in T cell activation and utilize ATP to phosphorylate target molecules (104). Oxidative phosphorylation functions as the main energy sources during T cell activation (105), therefore the downregulation of the process may contribute to the inhibition of T cell activation during spaceflight. Interestingly, mitochondrial dysfunction has been suggested to be a cell intrinsic trigger for T cell exhaustion ( (106) T cell exhaustion is normally induced by chronic antigen stimulation, but during spaceflight microgravity has been suggested to induce activation of T cells that decreases over time (107). We propose that ROS from dysfunctional mitochondria induces gene expression changes by modulating key transcription factors such as HIF-1α and others as seen by the upregulation in the generic transcriptional pathway (**Figure 2**). T cell exhaustion is characterized by reduced effector functions, reduced cytokine secretion of IL-2, TNF-γ and TNF-α and upregulation of inhibitory receptors (108). Hallmark T cell exhaustion genes such as CTLA-4 (1.4, 0.004), TIGIT (1.6, 0.001), CD274 (1.9, 0.001, which encodes PD-L1), BCL6 (1.5, 0.004), BACH2 (1.4, 0.001) and EOMES (2.2, <0.001) were upregulated in our dataset. CTLA-4 and TIGIT are inhibitory receptors that become upregulated on the surface of exhausted T cells and serve to negatively regulate immune function. Similarly, CD274 serves as the ligand for inhibitory receptor PD-1, decreasing cytokine production (109). EOMES is a T-box transcription factor that drives T cell exhaustion by mediating the transcription of inhibitory receptors (110). Likewise, our dataset shows the downregulation of IL-10 and its subsequent signaling pathway. Research has correlated decreased IL10 (-4.2, 0.002) expression with an increased amount of exhausted CD8 T cells (111). Decreased cytokine secretion has been continuously noted in PBMCs from astronauts returning from space missions and in cells cultured in simulated microgravity conditions (102). Comparatively, our data shows the upregulation of all three subunits of the IL-2 receptor (IL2RA (1.5, 0.003), IL2RB (1.4, 0.007), IR2RG (1.3, 0.007)). The upregulations of these receptors may be due to an adaptive response from the lack of cytokine secretion. Furthermore, the loss of cytotoxic abilities in exhausted T cells aligns with the inability to engulf pathogens after spaceflight (**Table 3**). Equally, downregulation of cell migration and endocytosis (**Table 3**) may work cooperatively to reduce pathogen engulfment from activated T cells.

Emerging data has also implicated GPCRs with T cell exhaustion (112), supporting our claim that persistent GPCR stimulation reprograms T cells to an exhaustive like state in microgravity. In this referenced study, it was shown that G protein alpha S GPCRs were highly expressed in exhausted CD8 T cells. Most notably, ADORA2A (2.2, 0.002), GPR65 (1.7, <0.001) and FFAR (3.0, 0.002) were positively correlated with T cell exhaustion. Our data shows the upregulation of these GPCRs. Likewise, G alpha q/11 and G alpha S coupled GPCRs were shown to have the highest mean correlation with T cells dysfunction, implicating intracellular calcium and cAMP activation with T cell exhaustion. Therefore, we propose that inhibition of T cell activation during spaceflight is due to a T cell state that parallels features of T cell exhaustion.

## Conclusion

**Figure 4:**
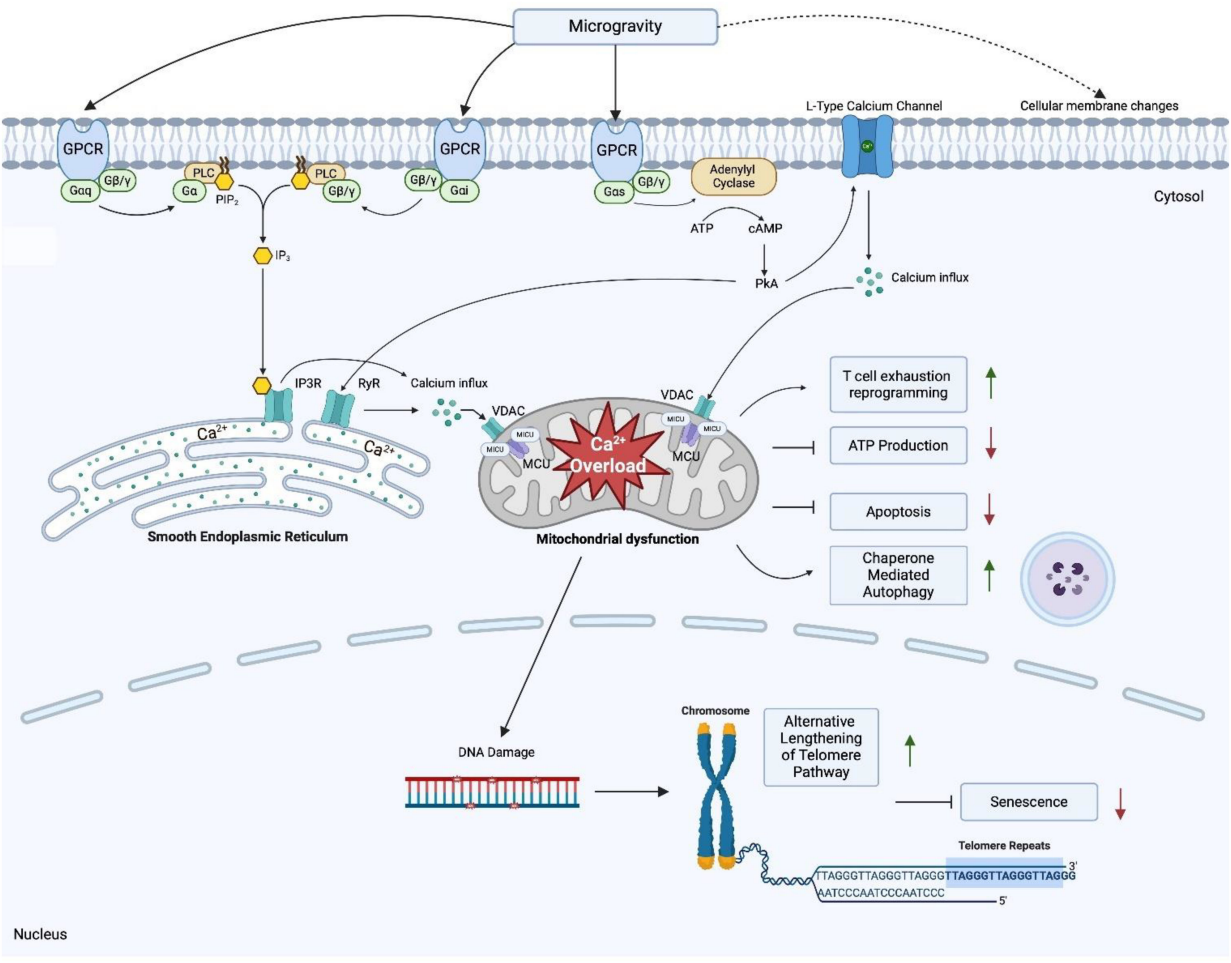
Proposed mechanisms underlying mitochondria dysfunction, T cell dysregulation and telomere elongation in space Created in BioRender. Jimenez-Chavez, L. (2025) https://BioRender.com/n91t558.

Our present study represents the most comprehensive transcriptomic dataset collected from 11 astronauts in space. The 2030 DEGs in our dataset for 11 astronauts after spending more than 135 days on the ISS provides insight to the mechanisms by which humans adapt to the unique space environment. Based on the 2030 DEGs of our dataset we hypothesize that spaceflight results in an increased influx of cytosolic calcium which ultimately causes mitochondrial dysfunction during spaceflight. We found dysregulations in GPCR related genes that point to enhanced calcium influx and mobilization into the cytosol and mitochondria. Our findings also indicate microgravity induced membrane disruption, further enhancing the influx of cytosolic calcium, and modulating GPCR activity. Low levels of cytosolic calcium are needed to regulate homeostasis and modulate cell function within eukaryotes, but increased concentrations can induce calcium overload in the mitochondria and impede cellular processes (113). Our dataset also suggests activated survival mechanisms in T cells, allowing them to bypass apoptotic and senescent cues, and explains telomere lengthening during spaceflight. Additionally, we uncover the mechanisms that inhibit T cell activation during and after spaceflight. The proposed mechanisms for mitochondria dysfunction, immune dysregulation and telomere elongation are shown in **Figure 4**.

We further hypothesize that, for other cell types, spaceflight induces mitochondrial dysfunction through calcium overload via microgravity induced pressure changes that upregulate GPCR-calcium signaling. In T lymphocytes this manifests as a T cell exhaustion-like state that is independent of antigen stimulation. All in all, while metabolically less active, these cells remain viable due to cell survival mechanisms that allow them to bypass senescence, apoptosis and undergo telomere elongation through the ALT pathway.

### Limitations

Due to the enormous complexity of human studies involving astronauts, the pre- and in-flight blood collection could not be synchronized for all astronauts, resulting in different exposure times to the space environment among the astronauts (135 to 210 days in the present study). Grouping the blood samples collected at different time points can generate “data noise” (114). During return of the inflight samples to the ground, the cells in the blood collection tube experienced changes from μg to hyper g (<1.6g) and then to 1g after landing. *In vitro* studies in which cultured blood cells experienced changes of gravity conditions may provide useful information to determine whether DEGs in our dataset were induced by reentry of the blood samples. It has been reported that the number of DEGs due to altered gravitational force is low, as 50 early responsive DEGs were found in T cells during a parabolic flight when comparing hyper-g or μg to 1g (115). Transferring T cells from μg to a 1g centrifuge on the ISS only identified 47 dysregulated genes (11). Gene expression changes in PBMC can also be impacted by the distribution of different subtypes of blood cells in space. The percentages of sub-blood cell types were not significantly altered in space in comparison to the pre-flight samples (Crucian, unpublished), in this case, it should have minimal impact on the DEGs found in the present study. The populations of different subtypes of blood cells were also similar between pre- and in-flight samples from computations of DEG using a deconvolution algorithm (Data not presented here). Based on these results, we believe that the majority of DEGs found in our study reflect a homeostatic response. The impact of these physical stressors can be eliminated using an RNA stabilizer after blood was drawn. However, RNA stabilizers were not used due to safety reasons. We also recognize that information generated from transcriptomics data needs to be verified in future studies.

## Data Availability

The datasets presented in this article are not readily available because of ethical and privacy restrictions per NASA guideline. Requests to access the datasets should be directed to the corresponding author. Aggregated data to understand and assess the conclusions of this research are available in the figures and tables including supplemental materials. Aggregated data (read count tables and metadata) have been deposited in NASA's Life Sciences Data Archives (LSDA). Investigators can request access to the astronaut data at jsc-lsda@mail.nasa.gov.

## Supplementary material

Supplementary Figure S1 Volcano plot of differentially expressed genes. The dashed lines represent fold change of 2 (in-flight vs pre-flight) and the p-value corresponding to FDR=0.05, respectively. The top 20 significantly DEGs (ranked by FDR) are labeled.

Supplementary Table S1 DEG associated with endocytosis function.

Supplementary Table S2 Gene ontology analysis using the GSEA method for cellular components (GOCC) and functions (GOMF).
