# Supplementary material for: Transcriptomics analysis reveals potential mechanisms underlying mitochondrial dysfunction and T cell exhaustion in astronauts’ blood cells in space
Source: Front Immunol. 2025 Jan 20;15:1512578. doi: 10.3389/fimmu.2024.1512578 (PMC11788081; doi:10.3389/fimmu.2024.1512578)
Supplement: Supplementary Figure S1 — Volcano plot of differentially expressed genes. The dashed lines represent fold change of 2 (in-flight vs pre-flight) and the p-value corresponding to FDR=0.05, respectively. The top 20 significantly DEGs (ranked by FDR) are labeled. [file Presentation1.pdf]

SUPPLEMENTAL MATERIALS

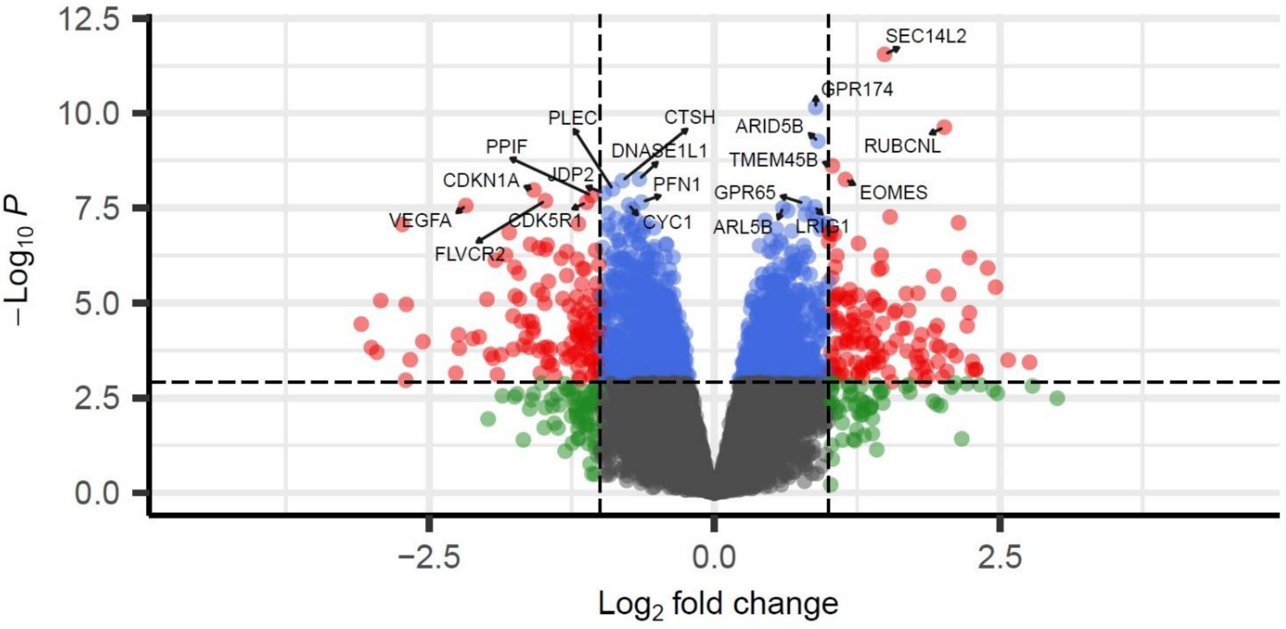

Figure S1. Volcano plot of differentially expressed genes. The dashed lines represent fold change of 2 (in-flight vs pre-flight) and the p-value corresponding to FDR=0.05, respectively. The top 20 significantly DEGs (ranked by FDR) are labeled.

Table S1. DEG associated with endocytosis function.

Gene	FC	FDR	Gene	FC	FDR	Gene	FC	FDR
CDKN1A	-3.0	0.000	ANXA5	-1.6	0.001	MEF2D	-1.4	0.004
PFN1	-1.6	0.000	EGR1	2.3	0.001	ATG2B	1.4	0.004
GSTP1	-1.7	0.000	PRKD2	1.6	0.001	RASA4	-1.3	0.004
MAP1S	-3.5	0.000	MARCO	-2.1	0.001	ABL1	-1.3	0.005
LILRB1	-1.8	0.000	BRAF	1.4	0.001	LRP1	-1.3	0.005
VIM	-1.6	0.000	ARPC3	-1.3	0.001	ATP6AP1	-1.2	0.005
ACTG1	-1.3	0.000	CAPG	-1.7	0.001	JAK1	1.3	0.005
LGALS3	-2.0	0.000	P2RX7	-1.4	0.001	FGD6	-1.3	0.005
KLF2	1.7	0.000	CHRNA7	-1.8	0.001	LDLRAD3	-2.3	0.005
CHN1	2.1	0.000	TREML2	2.6	0.001	VAMP8	-1.3	0.005
SIGLEC1	-3.8	0.000	AXL	-2.0	0.001	FCGR3B	3.6	0.005
PPT1	-1.5	0.000	ITGB2	-1.3	0.001	TNFSF10	1.8	0.005
STAB1	-1.7	0.000	PLCG2	1.4	0.002	ATP5B	-1.2	0.005
IRF8	-1.5	0.000	IL18	-1.5	0.002	IQSEC1	1.4	0.005
GRN	-1.6	0.000	GPR18	1.5	0.002	CDK2	1.4	0.005
CD151	-1.6	0.000	VAMP2	1.4	0.002	PTRHD1	-1.3	0.006
PKN2	1.5	0.000	APP	-1.3	0.002	SYK	-1.4	0.006
FCN1	-1.8	0.000	WIPF1	1.4	0.002	NPC1	1.4	0.006
HMOX1	-2.9	0.000	FCMR	1.5	0.002	CD36	-1.8	0.006
BRK1	-1.4	0.000	PTPRC	1.4	0.002	ANXA1	-1.4	0.006
AP2S1	-1.5	0.000	FPR1	2.1	0.002	IFNAR1	1.4	0.006
BTK	-1.4	0.000	JMJD6	-1.4	0.002	RALBP1	1.4	0.006
MGEA5	1.4	0.000	STX1A	-3.2	0.002	AFF2	2.0	0.006
RARA	-2.0	0.000	FCGR1A	-1.6	0.002	TREM1	1.9	0.007
CTNNB1	-2.0	0.000	CEACAM3	3.9	0.002	PARK7	-1.3	0.007
CANX	-1.3	0.000	TMEM165	-1.3	0.002	MET	-2.9	0.007
MAPKAPK3	-1.4	0.000	BIN1	-1.5	0.002	CD163	-1.8	0.007
PYCARD	-1.5	0.000	SLC35A2	1.5	0.002	TLR4	2.0	0.007
EZR	1.5	0.000	SERPINE1	-8.0	0.003	HSPG2	-2.0	0.007
C5	-1.8	0.000	RAB5C	-1.3	0.003	PIK3C2A	1.3	0.008
NME2	-1.4	0.000	ATP6V0C	-1.3	0.003	CLEC4G	-2.0	0.008
AP2M1	-1.3	0.000	DNM2	-1.2	0.003	RAB4A	-1.3	0.008
MERTK	-2.8	0.000	HRAS	-1.4	0.003	UBE2L3	-1.2	0.008
PAN3	1.3	0.001	PICK1	-1.4	0.003	MAPK14	1.6	0.008
SPHK1	-2.0	0.001	ATG2A	2.0	0.003	CD2AP	1.3	0.008
RORA	1.5	0.001	SLC7A11	1.7	0.003	CSF2RB	1.4	0.008
C3	-1.7	0.001	USPL1	-1.5	0.003	DENND1A	-1.3	0.008
RRAS2	1.5	0.001	LAMTOR2	-1.4	0.003	STK4	1.2	0.008
ZNF217	1.7	0.001	ITGAM	-1.3	0.003	LRP3	-1.6	0.008
ACTB	-1.4	0.001	WNK1	1.3	0.004	HIP1R	1.4	0.008
FAS	1.8	0.001	TMEM173	-1.3	0.004	EIF2AK1	-1.2	0.009
PKM	-1.3	0.001	RGCC	-2.1	0.004	BTBD9	1.2	0.009
RASA2	1.3	0.001	MYO1G	-1.2	0.004	PRNP	-1.3	0.009
LIPA	-1.6	0.001	LRPAP1	-1.3	0.004	TM2D3	1.2	0.009
CYBB	-1.8	0.001	ARPC4	-1.2	0.004	HPSE	-1.7	0.009
CALR	-1.3	0.001	PRKX	1.2	0.004	MEX3B	-1.9	0.010
DPYSL2	-1.9	0.001	RAC1	-1.4	0.004	RAB34	-1.4	0.010
TGFB1	-1.4	0.001	SMARCB1	-1.3	0.004	MEX3B	-1.9	0.010
RUBCN	1.4	0.001	ATP6V1F	-1.3	0.004	RAB34	-1.4	0.010

Table S2. Gene ontology analysis using the GSEA method for cellular components (GOCC) and functions (GOMF).

GOCC	FDR		GOMF	FDR
VACUOLAR_LUMEN	2.55E-08		MOLECULAR_FUNCTION_INHIBITOR_ACTIVITY	2.79E-03
COLLAGEN_CONTAINING_EXTRACELLULAR_MATRIX	2.55E-08		IMMUNE_RECEPTOR_ACTIVITY	5.22E-03
T_CELL_RECEPTOR_COMPLEX	1.57E-07		PEPTIDE_BINDING	5.59E-03
LYSOSOMAL_LUMEN	1.57E-07		MHC_CLASS_I_RECEPTOR_ACTIVITY	3.59E-02
EXTERNAL_ENCAPSULATING_STRUCTURE	2.37E-07		PROTON_CHANNEL_ACTIVITY	3.59E-02
MITOCHONDRIAL_PROTEIN_CONTAINING_COMPLEX	1.87E-06		PHOSPHATIDYLINOSITOL_BISPHOSPHATE_BINDING	3.59E-02
NUCLEOSOME	2.26E-06		ENDOPEPTIDASE_REGULATOR_ACTIVITY	3.59E-02
VESICLE_LUMEN	7.24E-06		PEPTIDASE_REGULATOR_ACTIVITY	3.59E-02
ENDOPLASMIC_RETICULUM_LUMEN	2.47E-05		G_PROTEIN_COUPLED_RECEPTOR_ACTIVITY	3.59E-02
MITOCHONDRIAL_MATRIX	2.04E-04		ENZYME_INHIBITOR_ACTIVITY	4.65E-02
